# Revisiting the use of web search data for stock market movements

**DOI:** 10.1038/s41598-019-50131-1

**Published:** 2019-09-18

**Authors:** Xu Zhong, Michael Raghib

**Affiliations:** IBM Research Australia, Melbourne, Victoria Australia

**Keywords:** Computational science, Information technology, Information theory and computation

## Abstract

Advances in Big Data make it possible to make short-term forecasts for market trends from previously unexplored sources. Trading strategies were recently developed by exploiting a link between the online search activity of certain terms semantically related to finance and market movements. Here we build on these earlier results by exploring a data-driven strategy which adaptively leverages the *Google Correlate* service and automatically chooses a new set of search terms for every trading decision. In a backtesting experiment run from 2008 to 2017 we obtained a 499% cumulative return which compares favourably with benchmark strategies. A crowdsourcing exercise reveals that the term selection process preferentially selects highly specific terms semantically related to finance (e.g. Wells Fargo Bank), which may capture the transient interests of investors, but at the cost of a shorter span of validity. The adaptive strategy quickly updates the set of search terms when a better combination is found, leading to more consistent predictability. We anticipate that this adaptive decision framework can be of value not only for financial applications, but also in other areas of computational social science, where linkages between facets of collective human behavior and online searches can be inferred from digital footprint data.

## Introduction

Advances in machine learning made possible by the availability of very large data sets generated by mobile devices, satellite images, distributed sensors and internet activity are driving a growing interest in the potential of Big Data to radically transform the investment decision process at financial institutions. Massive data sets containing the digital footprint of the interactions between people and the internet, such as *Wikipedia* access logs, *Twitter*, and web search traffic on *Google* and *Yahoo!*, offer a window into the collective behavior of millions. Correlations found between such data and real world outcomes are now routinely used for short term forecasts (or ‘nowcasts’) of great practical interest like movements of the stock market^1–4^, influenza epidemics^5–8^, consumer behavior^9^, and unemployment rates^10,11^.

*Google Trends* is a publicly available service that returns the normalized search volume for given terms within a time window and geography. This information, known as web search data, reflects the rapidly changing interests of millions of people as they go about the business of gathering information online. It has been shown that the search volume of certain terms that have a semantic relationship to finance can predict a number of variables of interest, such as stock transaction volume^12^, market volatility^13^ and liquidity^14^, stock returns^15,16^, as well as BitCoin price^17^. This finding has been used to develop automated stock trading strategies targeting market movements^1,18,19^ and risk^20,21^.

**Figure 1:**
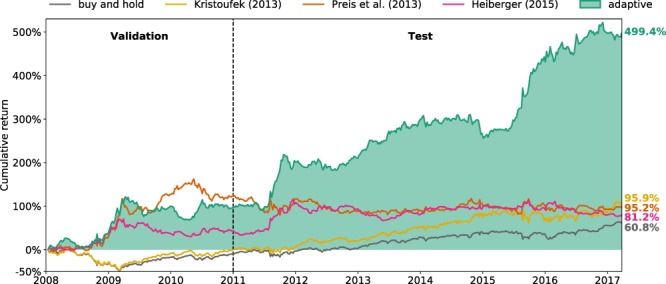
Comparison of the cumulative return of the adaptive trading strategy against the baseline buy and hold strategy and three benchmark strategies in a backtesting experiment running from January 6th, 2008 to March 26th, 2017. The experimental period is split into a validation period, used to tune the hyperparameters of our model, and a testing period. The baseline buy and hold strategy simply follows the market by buying the DJIA at the beginning of the experiment and holding the portfolio until the end. Kristoufek’s strategy follows the market with a mechanism allowing it to diversify risk using web search data, leading to a better performance than buy and hold. All the other strategies automatically long or short the whole portfolio or individual stocks on a weekly basis by analyzing web search data. The strategies proposed by Preis *et al*. and Heiberger are static; they use pre-defined search terms and fixed decision heuristics. Returns for these two strategies saturate after 2012, evidencing loss of predictability. By selecting different search terms and retraining a predictive model for every decision, the adaptive strategy increases the portfolio value by nearly 500% at the end of the experiment, which is 404% more than the most-profitable benchmark strategy (Kristoufek’s).

Seminal studies found that the search volume of certain terms semantically related to finance have predictive power on stock market movements, when compared with a broad range of other topics. The relatedness of some terms to finance, such as company names^18^ or tickers^20^, is sometimes based on common sense. Others have taken a quantitative approach to collate a set of terms belonging to a certain topic^1,22^. Once search terms are chosen, they remain fixed throughout the execution of the automated trading. This presupposes that the predictability of the initial set of terms remains constant over the long-term, which is problematic due to the non-stationary nature of financial time series. In fact, Curme *et al*. showed that the predictability of their search terms is limited to a period from 2006 to 2011^22^. We also found similar results in our experiment for the strategies based on fixed search terms proposed by Preis *et al*.^1^ and Heiberger^18^ (see Fig. 1). A common assumption across web-search-based strategies is based on Herbert Simon’s theory of decision-making^23^, where perceived uncertainty about a prospect triggers the decision-making process of individuals, which begins by gathering information. This leads to a decision heuristic where a relative increase in the search volume of these terms is a proxy for investor uncertainty, implying a higher level of risk and thus triggering a short position. On the other hand, decreasing search volume is considered a sign of investor confidence, triggering a long position. These strategies are appealingly explainable, but leave out of consideration other relationships that might possess better predictability properties.

In this paper we propose a strategy for predicting market movements with web search data by combining an adaptive approach with automatic search term selection. Adaptive methods for nowcasting based on web search data were introduced for influenza nowcasting in^6^. Their model was retrained every week on *Google Flu Trends* data points from the previous 16 weeks. The search terms used to derive the *Google Flu Trends* are occasionally updated by *Google* to respond to variation in search behaviors. Here we update the set of search terms much more frequently (weekly) by querying the *Google Correlate* service (GCS). GCS returns up to 100 search terms ranked by the Pearson correlation coefficient between their search volumes and a target time series. In our case, the target time series is the Dow Jones Industrial Average (DJIA) index within a fixed window before the trading week (see Methods). Because the companies in the DJIA are all based in the U.S., we restrict the statistics of search volumes to that geography. Rather than relying on heuristic relationships between search volume and market movements, we train a linear regression model that combines the search volumes of the terms returned by GCS to predict the DJIA for the next two weeks, where we manage overfitting by curating the search terms returned by GCS using an automated feature selection technique.

Unfortunately, the GCS only provides web search data until March 12th, 2017, which prevents the acquisition of the correlated search terms and their high-precision search volumes after that date. Our experiments use all the data that can be accessed from the GCS and demonstrate the potential of adaptive and quantitative selection of search terms for predicting market movements. The growing interest in finding correlations in web search data, of which this paper is an exemplar, may create incentives for other search engines to provide similar functionality in the near future.

## Results

To evaluate the performance of our adaptive strategy, we replicate the trading experiment proposed by Preis *et al*.^1^ within a more recent time span (January 6th, 2008 to March 26th, 2017). In this experiment, we execute one of two available trading options at the end of each week *t*:

1. **long**: buying the DJIA at the closing price *P* of the first trading day of week *t* + 1 and selling the DJIA at the closing price of the first trading day of week *t* + 2; or
2. **short**: selling the DJIA at the closing price of the first trading day of week *t* + 1 and buying the DJIA at the closing price of the first trading day of week *t* + 2.

If our regression model predicts an increasing trend of the DJIA from *t* + 1 to *t* + 2, we execute the long option, which changes the portfolio value *V* at *t* + 2 to $V(t+2)=V(t+1)P(t+2)/P(t+1)$. Otherwise, we execute the short option and get a portfolio of value $V(t+1)P(t+1)/P(t+2)$ at *t* + 2.

Figure 1 compares the performance of our adaptive strategy with the baseline buy and hold strategy as well as three benchmark strategies based on web search data:

1. Preis *et al*.^1^: using debt, the best-performing search term found among the 98 semantically different keywords studied in^1^ to trade the DJIA.
2. Kristoufek (2013)^20^: using tickers of companies (e.g. XOM) as search terms to diversify portfolio among stocks in the DJIA.
3. Heiberger (2015)^18^: using company names (e.g. ExxonMobil) as search terms to trade individual stocks in the DJIA.

Implementation details of these three strategies are described in Methods. The baseline buy and hold strategy is outperformed by all the other strategies. It only achieves 61% final cumulative return (final portfolio value/initial portfolio value - 1), since it simply follows the market. Kristoufek’s strategy also follows the market, but by dynamically diversifying the portfolio using web search data, it yields a higher final cumulative return (96%). A limitation of Kristoufek’s strategy is the lack of a mechanism allowing it to leave a bearish market, which makes it vulnerable to market downturns. The strategy developed by Preis *et al*. leaves (enters) the market when an increasing (decreasing) level of risk is predicted, which led to a 327% cumulative return from 2004 to 2013^1^. This strategy is effective within a comparatively narrow period of time (late 2008–mid 2010) relative to the time span of our experiment, yielding a 95% return overall. Heiberger’s strategy is also effective within two relatively narrow periods (late 2008–early 2009 and mid 2011–early 2012), resulting in a 81% return at the end of the experiment. Our adaptive strategy considerably outperforms the benchmarks, generating close to 500% final cumulative return, which is 404% more than the most-profitable benchmark strategy (Kristoufek’s).

Table 1 compares the mean, standard deviation (STD), and Sharpe ratio of the weekly return ($V(t+1)/V(t)-1$) of the adaptive strategy, the baseline strategy, and the benchmark strategies. The adaptive strategy generates the highest mean weekly return, which leads to the highest cumulative return as shown in Fig. 1. The STD of weekly return is a measure of variability and therefore risk. Heiberger’s strategy trades individual stocks separately, which helps diversity the risk and leads to the widest portfolio breadth and lowest risk. The Sharpe ratio is a commonly used measure of risk-adjusted portfolio performance, which penalizes high risk. The adaptive strategy generates a Sharpe ratio that is over two times that of any other strategy.

**Table 1:** Mean, standard deviation (STD) and Sharpe ratio of the weekly return $V(t+1)/V(t)-1$ of the adaptive strategy versus other strategies.

Strategy	Mean weekly return	STD of weekly return	Sharpe ratio
Buy and hold	0.13%	2.53%	0.05
Kristoufek (2013)	0.19%	3.17%	0.06
Preis *et al*. (2013)	0.17%	2.56%	0.07
Heiberger (2015)	0.14%	**2.02%**	0.07
**Adaptive**	**0.41%**	2.54%	**0.16**

The third column shows the STD of weekly return, which is a measure of variability and therefore risk. The fourth column shows the Sharpe ratio, a measure of portfolio performance, which adjusts returns by penalizing large variance. The adaptive strategy shows a Sharpe ratio that is over two times as large as that of any other strategy.

To test whether the performance of the strategies based on web search data might be due to chance, we follow the same approach originally suggested in this context by Preis *et al*.^1^, whereby we randomize the strategies by replacing the trading decisions with uncorrelated random choices. For Kristoufek (2013), we uniformly shuffle the portfolio allocation across the stocks in the DJIA. For Preis *et al*. (2013), Heiberger (2015), and the adaptive strategy, long or short options are executed with the same probability. We compute the kernel density estimate (KDE), with a Gaussian kernel and bandwidth calculated with Silverman’s rule of thumb, of the final portfolio value from 10,000 independent realizations of each randomized strategy. The probability that a better performance can be obtained by chance rather than trading using web search data is calculated from the KDE and listed in Table 2. The probability that a higher final portfolio value than the adaptive strategy can be obtained by chance is only 9.08e-04. This probability is at least two orders of magnitude smaller than the results of the benchmark strategies.

**Table 2:** Probability of outperforming a trading strategy by chance.

Strategy	Probability of being outperformed by chance
Kristoufek (2013)	2.45e-1
Preis *et al*. (2013)	1.17e-01
Heiberger (2015)	1.21e-01
**Adaptive**	**9.08 × 10** ^**−4**^

We compare the performance of the trading strategies that are based on web search data with their corresponding randomized strategy, i.e., replacing trading decisions with uncorrelated random choices. The KDEs of the final portfolio value of the randomized strategies are computed from 10,000 independent realizations. From the KDE we calculate the probability that a better performance than trading using web search data can be obtained by chance. The probability that the performance of the adaptive strategy is due to chance is extremely small (9.08e-4). When compared with other strategies, this probability is at least two orders of magnitude smaller.

**Figure 2:**
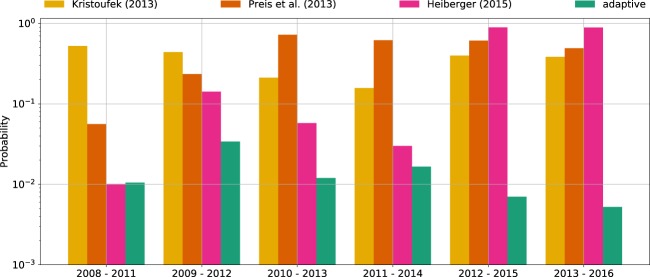
Effect of changing time window on predictability, measured as the probability of being outperformed by chance (lower probability implies higher predictability). We use the approach described in the caption of Table 2, but compute the probability in six overlapping 4-year windows instead of the whole time span of the experiment (9 years 3 months). The predictability of the benchmark strategies varies as the window moves, whereas the adaptive strategy has consistently high predictability.

Curme *et al*. used overlapping windows to analyze changes in the performance of the trading strategy they proposed^22^. We follow this approach to study the temporal variations in the predictability of a given trading strategy. As the window moves forward, loss of predictability can be identified by an increase of the probability that a better return can be obtained by chance. Figure 2 depicts this probability for the benchmark strategies together with the adaptive strategy computed in six overlapping 4-year windows (adjacent windows overlap by three years). The benchmark strategies show high predictability only for some specific time windows. On the other hand, the adaptive strategy consistently shows high predictability across all windows.

**Figure 3:**
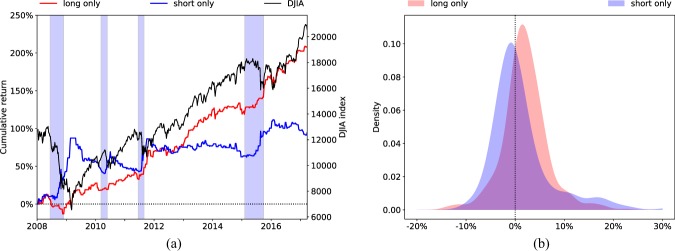
Contributions to cumulative return due to the long and short decisions of the adaptive strategy. When quantifying the contribution of long decisions, we implement the adaptive strategy in such a way that we execute long decisions when recommended but never execute short decisions. Conversely, when evaluating the contribution of short decisions, we take short decisions only but never take long decisions. (**a**) Cumulative return (left axis) for long only and short only decisions and the DJIA (right axis). (**b**) The KDE of the returns in overlapping eight-week windows, obtained by long only and and short only decisions. The long decisions generate smaller but more steady returns, compared to the short decisions. The short decisions are better at dealing with the four sustained market downturns illustrated by the blue bars in (**a**), which correspond to the Global Financial Crisis in 2008, the European Debt Crisis in 2010 and 2011, and the 2015 stock market selloff.

In order to quantify the relative contributions to portfolio returns of long and short decisions, we modify the adaptive strategy in the way proposed in^1^ where we only execute either long or short decisions. For instance, when evaluating the contributions of short decisions, we take short decisions only and ignore long decisions. Figure 3(a) illustrates the cumulative return obtained by the long only and short only versions of the adaptive strategy. Both long and short decisions increase portfolio value, but their contributions to portfolio performance are markedly different. Long decisions generate small but steady returns when compared to short decisions. In contrast, the role of short decisions seems to be detecting sustained downturns of the DJIA. This is suggested by the large positive returns observed during four short periods (see blue bars in Fig. 3(a)), when the strategy opted for leaving the market coinciding with four sustained market downturns: the Global Financial Crisis in 2008, the European Debt Crisis in 2010 and 2011, and the 2015 stock market selloff. The difference can also be seen in the two KDEs for long only and short only returns in overlapping 8-week windows (Fig. 3(b)). The short only returns are considerably more skewed to the right ($skewness=1.9$) than the long only returns ($skewness=0.3$), which indicates a higher probability of obtaining larger returns. Long decisions lead to larger average returns (2.0%) and smaller STD (4.6% than short decisions (*mean* = 1.2%, *STD* = 6.3). This implies a more steady and lower-risk growth in portfolio value.

**Figure 4:**
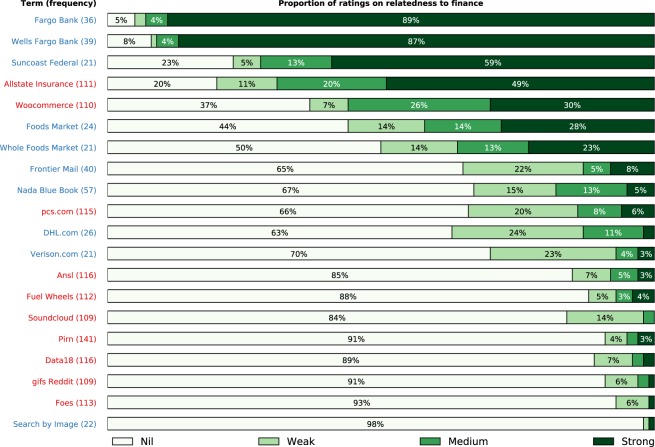
The ten most frequently selected terms (blue) and the ten most frequently rejected terms (red) from the automated term selection process, presented in descending order of relatedness to finance. Selection or rejection frequency is given in the parentheses on the right of the terms. The rankings are the aggregated ratings of ‘nil’, ‘weak’, ‘medium’, or ‘strong’ assigned to each of the terms by 100 workers on the *Figure Eight* crowdsourcing platform. The horizontal bars show the percentage of the four ratings for each of the terms. Nine out of the ten most frequently selected terms have a higher ranking of relatedness to finance than seven of the most frequently rejected terms. This seems to indicate that the term selection process is biased towards finance related terms.

To manage overfitting, our adaptive strategy reduces the dimensionality of the model by automatically selecting a subset of the search terms returned by GCS prior to model training (see Methods). It was discovered in^1,22^ that trading with terms that are more semantically related to finance achieved more successful outcomes. We categorize the relatedness to finance of the 10 terms most frequently selected/rejected by our feature selection process (shown in Fig. 4) using the crowdsourcing approach introduced in^22^. We set up a crowdsourcing job on the *Figure Eight* platform, where 100 workers rate the relatedness to finance of each term as ‘nil’, ‘weak’, ‘medium’, or ‘strong’. As we restrict our GSC query to the U.S., we made the crowdsourcing job only available to that geography. More details of the job are available in SI. The proportion of the ratings are illustrated in the horizontal bars in Fig. 4. We assign ‘nil’, ‘weak’, ‘medium’, and ‘strong’ ratings with scores of 0, 1, 2, and 3, respectively. The terms in Fig. 4 are presented in descending order of the total score of the 100 ratings. Nine out of the ten most frequently selected terms have a higher score than seven of the most frequently rejected terms. The most frequently selected terms received over 200 more ‘strong’ ratings and over 250 less ‘nil’ ratings than the most frequently rejected terms (see SI Fig. S2). These results seem to indicate that the term selection process is biased towards finance related terms, despite the fact that the term selection process does not take the semantics of the terms into consideration.

## Discussion

Our approach provides an independent replication of the finding by Preis *et al*.^1^ that the search volumes of certain terms semantically related to finance appear to have predictive power when used as a proxy for stock movements. We also speculate a possible trade-off between how semantically general a term is and its span of effectiveness. The adaptive strategy presented in this paper seems to be biased towards semantically specific terms (e.g., Wells Fargo Bank and Suncoast Federal), relative to the terms studied in^1^ (e.g., debt and economics). We think more specific terms, being idiosyncratic in nature, can better capture the transient interests of investors at the cost of a shorter span of validity. The reason why the adaptive strategy delivers persistent effectiveness is that it constantly monitors the predictive power of the current set of search terms with historical data, which are quickly replaced when better ones are found.

To compare with existing trading strategies that are developed based on web search data, we adopt the same setup as the trading experiment first proposed in^1^. This trading experiment does not account for transaction fees, dividends or capital gain taxes, which will affect the return when put into practice. The behavior of the adaptive strategy in a more realistic setting will be explored in the future.

The adaptive strategy is independently validated on an individual stock (IBM), where a higher return (234.6%) is obtained compared to other benchmark strategies (see SI Fig. S5). This provides additional evidence on the effectiveness of the adaptive strategy. We hope these encouraging results will motivate the exploration of the adaptive strategy in other settings. For example, the DJIA consists of companies based in the U.S., we therefore restricted the exploration of the adaptive strategy within that geography. This work can be naturally extended to other geographies like the FTSE 100 in the U.K. and the EURONEXT 100 in Europe; as well as commodity indices like the Thomson Reuters/CoreCommodity CRB Index. Further non-trivial extensions include the exploration of different combinations of machine learning and feature selection methods. In addition, this adaptive framework can naturally be extended to the nowcasting of other time series which might be inferred by proxy from digital footprint data.

As discussed in^24^, changes in the backend of the search engines as well as the algorithm that computes the statistics of the searches can lead to unexpected outcomes for decisions solely based on web search data. For instance, new versions of search engines now include autocomplete search suggestions, which may nudge user behaviors and interfere with the temporal evolution of the search volume of certain terms. We anticipate these changes will have less of an effect on our adaptive strategy than those based on fixed search terms, as we refresh the search terms before every decision.

In contrast to strategies based on fundamental analysis, our approach does not provide financial justification for the long or short decisions other than the correlation found between web search volume and the market index. However, we did find that the model-based feature selection algorithm preferentially picks terms semantically related to finance. We anticipate an in-depth study of the origin of this bias coupled with an ontology might provide a basis for explainability in the future.

Due to regulatory constraints, understanding the reliability of alternative data for financial decision making is essential to its integration with conventional sources of financial information. The reliability of data-driven decision models is usually understood in terms of the causal connection between features of the data and outcomes of decisions. This facilitates human-understandable explainability for decision makers to manage risk. However, reliability can also be interpreted as consistent predictability and high performance even in the absence of human-understandable explainability. In this paper, we tested three benchmark strategies which have higher explainability relative to our adaptive strategy, as they were based on search terms with apparent semantic relatedness to finance and established theories of decision making. However, we observed that the performance of these strategies varies significantly over time, which makes them less reliable. On the other hand, our adaptive strategy does not include semantic analysis on the search terms, which makes it challenging to justify the causal relationships between data and decisions. Nevertheless, it does have considerably more consistent predictability, which makes it useful when the risk profile of an investment requires mainly consistent predictability rather than a clear causal relationship between data and outcomes.

## Methods

### Adaptive strategy

Let *t* be the current week. We use the GCS to find the search terms (up to 100 terms) for which the search volume from *t* − *w* − 1 to *t* − 2 has the strongest correlation with the DJIA from *t* − *w* + 1 to *t*. The GCS also returns the search volumes of the search terms. Unlike *Google Trends*, which provides integer-based search volume data that show slightly different results for the same query, the GCS returns search volumes in deterministic floating numbers. Hence we use the GCS data in our experiment for replicability. We explored four values of *w* (52, 104, 156, and 208) and found that *w* = 208 provides the best return in our validation period (see SI Fig. S4). By training a linear regression model with the search volumes from *t* − *w* − 1 to *t* − 2 as input and the DJIA from *t* − *w* + 1 to *t* as output, we can predict the DJIA at *t* + 1 and *t* + 2 using the search volumes at *t* − 1 and *t*, respectively. A diagram of the workflow of the adaptive strategy is shown in SI Fig. S3.

The training data for each trading decision consists of 208 samples, each of which has up to 100 features (i.e., the search volumes of the terms returned by GCS). Thus, this task carries a high risk of overfitting due to the combination of high dimensionality and small sample size. We take two approaches to manage overfitting. First, we choose a linear regression model as the predictor, which is more robust to overfitting than non-linear models. Second, we curate the search terms returned by GCS using the recursive feature elimination (RFE)^25^ technique to reduce dimensionality. In RFE, the linear regression model is initially trained with all the features, where each feature is assigned a weight. A higher absolute value of a weight implies a higher contribution of that feature to the output of the model. Then the feature with the smallest contribution is removed, and a new linear regression model is trained with the remaining features. This process is recursively iterated until reaching the desired number of features to select. This number is automatically decided by minimizing the mean absolute error of the predicted weekly changes of the DJIA in a *K*-fold cross-validation with the training data. Here, we use the 10-fold cross-validation rule-of-thumb, which has proven useful in practice for small sets of training data^26^. We found that the curation process tends to select a rather small number of terms, thus effectively managing overfitting by keeping dimensionality low (see SI Fig. S1).

At every trading week *t*, if the linear regression model, trained with the final set of features, predicts an increasing trend from *t* + 1 to *t* + 2, we take a long position on the DJIA from *t* + 1 to *t* + 2. Otherwise, we take a short position from *t* + 1 to *t* + 2.

### Implementation of benchmark strategies

We implement the strategy proposed by Pries *et al*. (2013) with the optimal configuration found in^1^. The relative change of the search volume *G*(*t*) of the term debt is calculated as

1 $$\Delta G(t)=G(t)-\frac{1}{\Delta t}\mathop{\sum }\limits_{i=1}^{\Delta t}\,G(t-i),$$

where Δ*t* is set to 3. If Δ*G*(*t*) > 0, we take a short position on the DJIA from *t* + 1 to *t* + 2. Otherwise, we take a long position from *t* + 1 to *t* + 2.

We implement the strategy proposed by Heiberger (2015)^18^ with the following modifications. This strategy is originally based on the individual company names in the S&P 100. We adapted it to the DJIA components as of March 19th, 2015. In addition, we excluded Visa from the experiment as the initial public offering (IPO) of Visa occurred at a date later (March 18th, 2008) than the beginning of the experiment. The relative change of the search volume of the company names and the DJIA is calculated by Eq. (1), where Δ*t* is set to 3.

The strategy proposed by Kristoufek (2013)^20^ is based on the tickers of the components in the DJIA. We implemented it with the same structure of the DJIA as in Heiberger (2015). As in^20^ 12 tickers (BA, CAT, KO, DD, MCD, PG, HD, TRV, UNH, VZ, V, and DIS) are removed due to either too infrequent searches or ambiguity with stock unrelated terms and abbreviations. For a given week *t*, the weight *w*_*i*_(*t*) of a stock *i* in the portfolio is calculated as

2 $${w}_{i}(t)=\frac{{G}_{i}{(t)}^{-\alpha }}{{\sum }_{j=1}^{N}\,{G}_{j}{(t)}^{-\alpha }},$$

where *G*_*i*_(*t*) is the search volume of the ticker of stock *i* in week *t*; *N* is the total number of stocks in the portfolio; and the exponent *α* controls the strength of the impact of search volume on portfolio diversification, which is set to the value (0.6) that maximizes the Sharpe ratio in^20^. Equation (2) compares the search volumes among the tickers. When the *Google Trends* service is queried with multiple terms, the search volumes of the terms are normalized by the term that is most frequently searched. Hence for each ticker, we query the *Google Trends* service alongside the ticker that has the highest search volume (i.e., GE). This normalizes the search volume of the tickers and ensures the search volumes to be comparable.

## Supplementary information

Supporting information
