# Supplementary material for: Revisiting the use of web search data for stock market movements
Source: Sci Rep. 2019 Sep 18;9:13511. doi: 10.1038/s41598-019-50131-1 (PMC6751183; doi:10.1038/s41598-019-50131-1)
Supplement: Supplementary file 1 — Supporting information [file 41598_2019_50131_MOESM1_ESM.pdf]

Revisiting the use of web search data for stock market movements - Supporting Information

Xu Zhong, Michael Raghib

1 Number of terms selected

A total of 480 weekly decisions were made in our experiment, which ran from January 6th, 2008 to March 26th, 2017. For every decision, the Google Correlate™ service returned 100 search terms, which were automatically curated using the recursive feature elimination technique (see **Methods** in the paper for more details).

Fig. S1 shows the histogram of the number of terms selected by the curation process. Over 50% of the decisions are based on the search volume of just one or two terms; moreover 77% of the decisions are made using less than 5 search terms. The curation process never selected more than 20 terms.

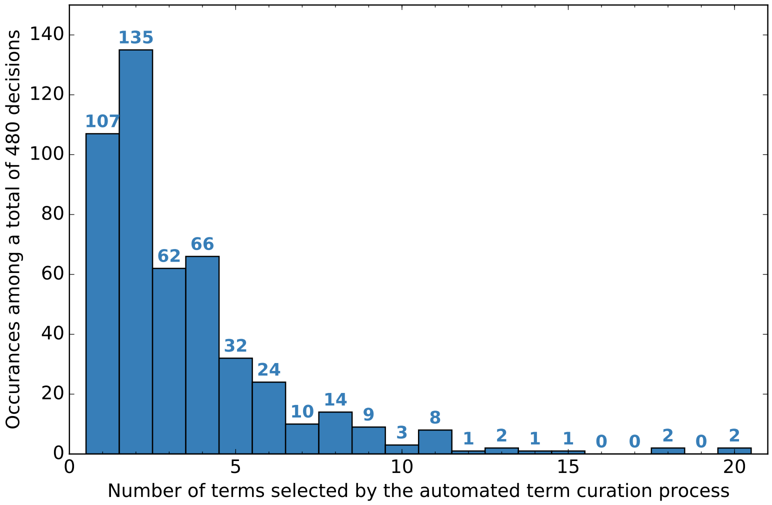

Figure S1. Occurrence of the number of search terms selected by the automated curation process from the 100 terms returned by the Google Correlate™ service.

2 Semantic relatedness to finance

To investigate if there is any semantic difference between the terms selected and rejected by the curation process, we set up a crowdsourcing job on the Figure Eight® platform[1]. We asked the workers located in the U.S. to rate the relatedness to finance of the 10 most frequently selected terms and the 10 most frequently rejected ones into one of four categories; namely, 'nil', 'weak', 'medium', or 'strong'. To rule out unreliable responses, we mixed the 20 original terms with a set of 10 quality control terms, consisting of 5 terms with an obviously strong relatedness to finance (i.e., 'Wall Street', 'Hedge Fund', 'Foreign Exchange', 'Financial Crisis', 'Stock Price') and 5 terms with apparently nil connection to finance (i.e., 'Name', 'Traffic Light', 'Ocean', 'Tree', 'Cat'). The correct rating for the former 5 terms and the latter 5 terms is 'strong' and 'nil', respectively. We collated 100 rating for each of the 20 original terms from workers who correctly rated at least 8 out of the 10 quality control terms.

Fig. S2 shows the number of ratings assigned to each of the four categories of relatedness to finance for the 10 most frequently selected terms (blue) and the 10 most frequently rejected ones (red). The 10 most frequently selected terms received over 200 more 'strong' ratings and over 250 less 'nil' ratings than the 10 most frequently rejected ones.

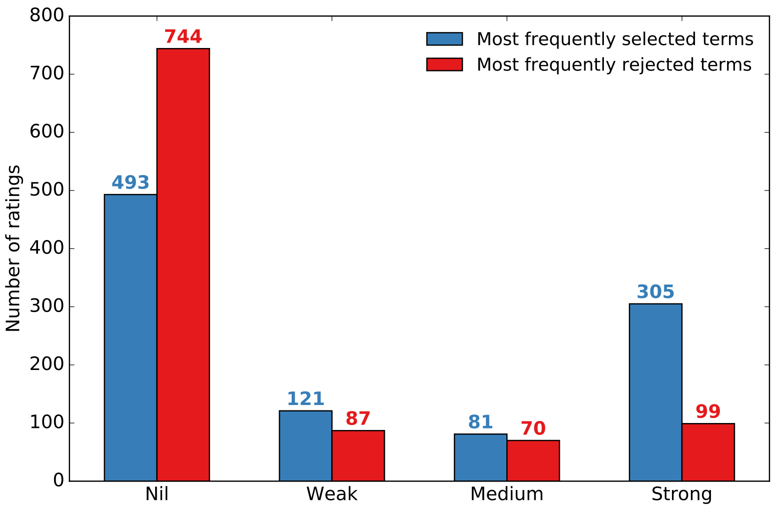

Figure S2. The total number of 'nil', 'weak', 'medium', and 'strong' ratings received by the 10 most frequently selected terms and the 10 most frequently rejected terms.

[1] https://www.figure-eight.com

3 Adaptive trading strategy

Fig. S3 shows the diagram of the adaptive trading strategy, illustrating the window used to query the search terms and search volumes from the GCS and train the linear regression model, and the long/short decision-making criteria. The following steps are repeated prior to each trading decision:

1. Acquisition of a new batch of web search data from the GCS,
2. Retraining the linear regression model with the new batch of web search data,
3. Predicting index movements.

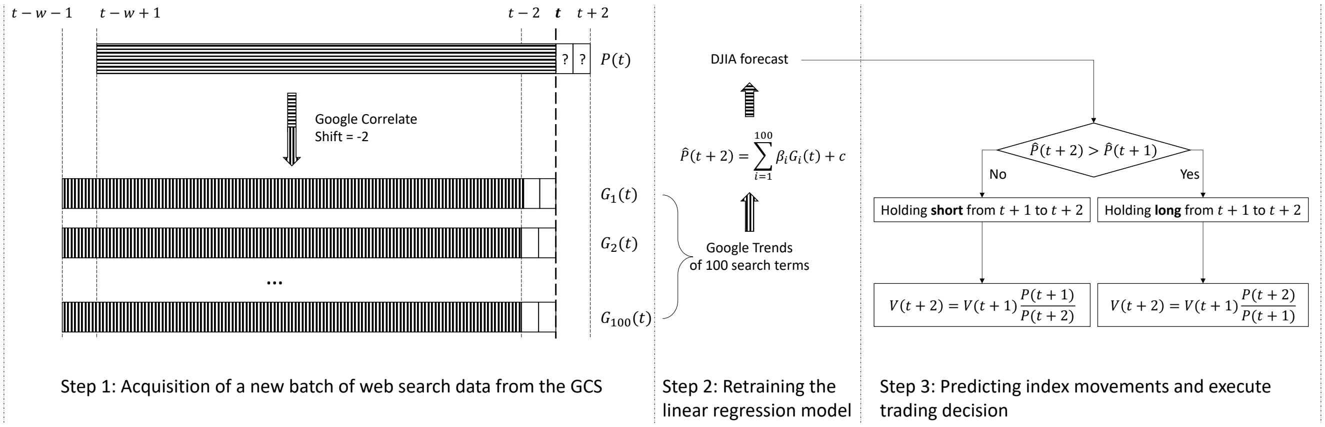

Figure S3. Diagram of the adaptive trading strategy.

4 Selection of window size

The window size in our adaptive strategy controls the length of historical data used to query the GCS and train the linear regression model. We split our data into a validation period (January 20 2008 to January 1 2011) and a testing period (January 1 2011 to March 26 2017). The validation period is used to select the optimal window size from four discrete values (52, 104, 156, and 208 weeks). Fig. S4 illustrates the effect of window size on the cumulative return at the end of the validation period. The window size of 208 weeks yields the optimal validation performance, and is used in the trading in the testing period.

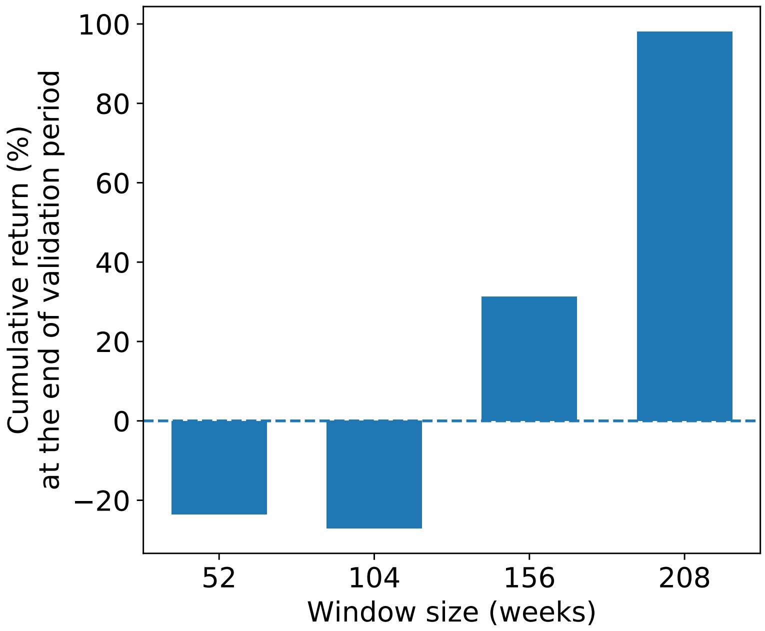

Figure S4. Effect of window size on the portfolio value at the end of the validation period.

5 Independent validation on an individual stock

The adaptive strategy, with the same parameters used in the trading experiment on the DJIA, is applied to trade an individual stock (IBM) as an independent validation. The benchmark strategies proposed by Preis et al.[1] and Heiberger[2] are also replicated on the IBM stock, again with the same setting used in the trading experiment on the DJIA. The benchmark strategy proposed by Kristoufek[3] is not considered in this experiment, since it diversifies a portfolio and therefore is not suitable for direct application to an individual stock.

Fig. S5 illustrates the cumulative return from January 20 2008 to March 26 2017. The adaptive strategy outperforms by a comfortable margin both the buy-and-hold baseline and the benchmark strategies, obtaining a 234.6% return at the end of the experiment. In contrast, the best performing benchmark strategy (Heiberger) obtains a 75.6% return.

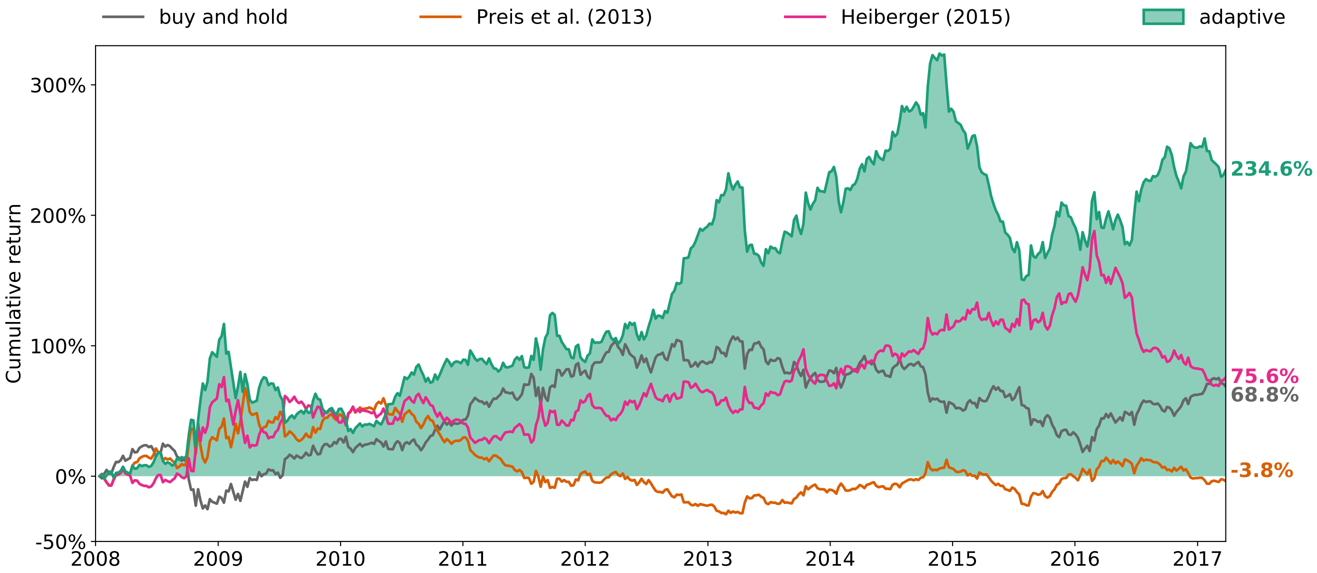

Figure S5. Validating the adaptive strategy on an individual stock (IBM), with the same parameters used in the trading experiment on the DJIA.

References

1. Preis, T., Moat, H. S. & Stanley, H. E. Quantifying trading behavior in financial markets using Google Trends. *Scientific reports* **3**, srep01684 (2013).
2. Heiberger, R. H. Collective attention and stock prices: Evidence from Google Trends data on Standard and Poor's 100. *PloS one* **10**, e0135311 (2015).
3. Kristoufek, L. Can Google Trends search queries contribute to risk diversification? *Scientific reports* **3** (2013).
